# Radiofrequency balloon ablation: 1-year outcomes of the AURORA study

**DOI:** 10.1007/s10840-024-01938-0

**Published:** 2024-11-08

**Authors:** Ilaria My, Boris Schmidt, Laura Rottner, Shota Tohoku, Marc Lemoine, David Schaack, Fabian Moser, Lukas Urbanek, Julius Obergassel, Djemail Ismaili, Jun Hirokami, Paulus Kirchhof, Karin Plank, Bruno Reissmann, Feifan Ouyang, Andreas Rillig, Julian Chun, Andreas Metzner, Stefano Bordignon

**Affiliations:** 1 Department of Cardiology, University Heart & Vascular Center Hamburg, University Medical Center Hamburg-Eppendorf, Martinistraße 52, 20246 Hamburg, Germany; 2 Cardioangiologisches Centrum Bethanien, Frankfurt am Main, Germany; 3 Universitätsklinikum Frankfurt, Med. Klinik 3, Kardiologie, Frankfurt am Main, Germany; 4 Institute of Cardiovascular Sciences, University of Birmingham, Birmingham, UK

**Keywords:** Atrial fibrillation, Pulmonary vein isolation, Thermal energy, Radiofrequency balloon, Single-shot isolation

## Abstract

**Background:**

A novel irrigated radiofrequency balloon (RFB) for pulmonary vein isolation (PVI) integrated into a 3D mapping platform was recently launched.

**Methods:**

Patients undergoing a first atrial fibrillation (AF) ablation at two German high-volume EP centers were included into the prospective AURORA registry. All patients underwent clinical follow-up (FU) at 90, 180, and 360 days following ablation including 48-h Holter ECGs.

**Results:**

A total of 99 patients were enrolled (43/99 (43.4%) women, median age 67 years (interquartile range [IQR] 59–74), 43/99 (43.4%) persistent AF (Pers-AF), median left ventricular ejection fraction (LVEF) 60% (IQR 62–55)). Eighty-eight patients completed the follow-up. Acute PVI was achieved in 383/383 (100%) PV. Single-shot PVI was achieved in 211/383 (55.1%) PVs. Primary adverse events occurred in 3% of patients (1 postprocedural pharyngeal bleeding, 1 myocardial infarction, 1 non-cardiovascular death); no pericardial effusion, stroke, or phrenic nerve paralysis was observed. Median ablation and procedure times were 23 (IQR 18–32) and 67 (IQR 57–85) min, respectively. Median dose area product was 761 (IQR 509–1534) mGycm^2^. AF-free survival after a median FU of 361 (IQR 261–375) days was 78.4% for paroxysmal AF (PAF) and 75.4% for Pers-AF (*p* value = 0.828). Early recurrence of atrial tachyarrhythmia at the 90-day visit was the only independent predictor for AF recurrence at 1 year upon multiple regression analysis (hazard ratio [HR] 3.198; 95% confidence interval [95% CI] 1.036–10.32, *p* value = 0.0433).

**Conclusion:**

RFB-based PVI is acutely successful, appears safe, and has comparable rhythm outcomes to other single-shot AF ablation tools. A recurrence of AF at 90 days predicts later AF recurrence.

**Graphical Abstract:**

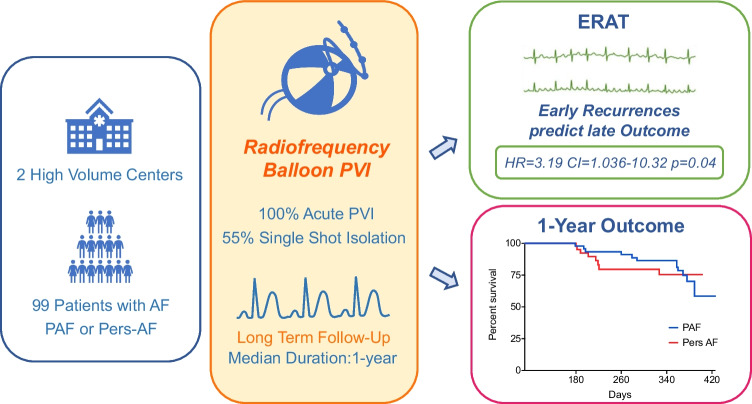

## Introduction

Incidence and prevalence of atrial fibrillation (AF) are increasing as a consequence of the aging demographics of the general population [1]. Rhythm control can improve AF-related symptoms and reduce AF-related outcomes such as stroke, heart failure events, and cardiovascular death [2]. AF ablation maintains sinus rhythm more effectively than antiarrhythmic drugs [3]. Pulmonary vein isolation (PVI) represents the key ablation target during AF ablation procedures [4].

Conventionally, PVI is achieved using point-by-point delivery of radiofrequency (RF) energy guided by a 3D mapping system. In response to the growing demand for ablation procedures, various novel technologies based on diverse energy forms and catheter configurations such as balloon-based systems are continually being introduced to the market. The goal is to enhance the efficacy and safety of PVI procedures, concurrently reducing its technical complexity and improving reproducibility among different operators.

**Fig. 1:**
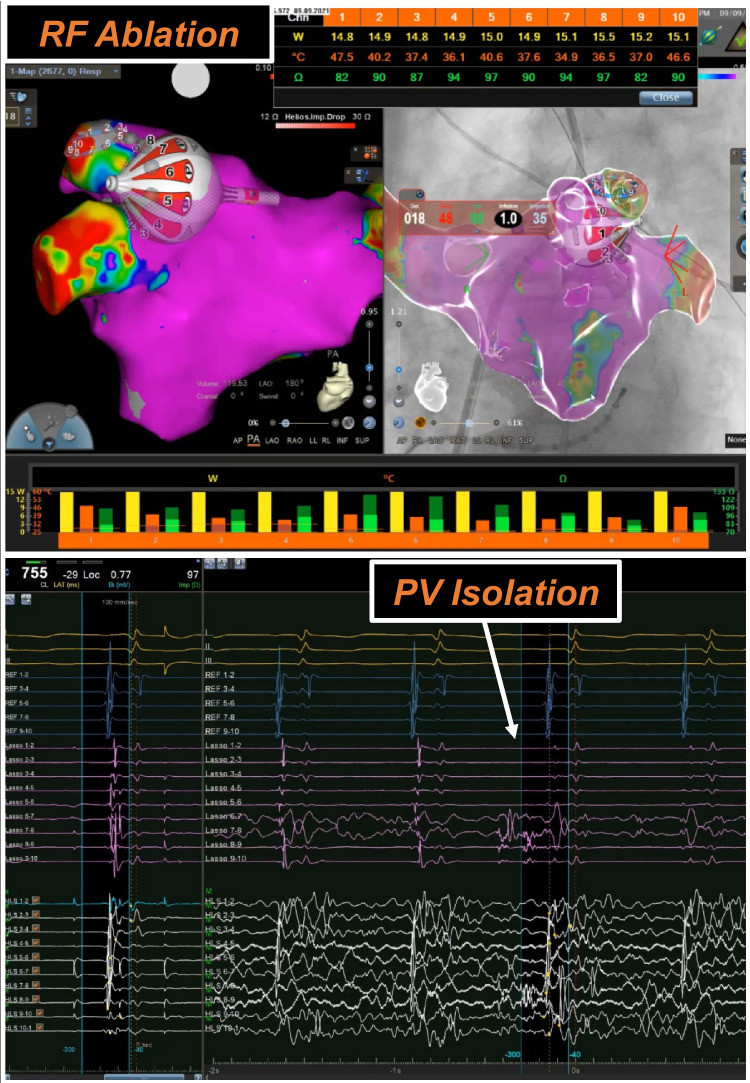
Radiofrequency balloon. HELIOSTAR radiofrequency balloon ablation visualized in CARTO 3 (Biosense Webster, CA, USA) (upper panel). Pulmonary vein signals are shown during the radiofrequency application along the left superior pulmonary vein (lower panel). During the ablation, real-time temperature and impedance values are shown simultaneously in the table and the respective graph bar (upper panel)

One such tool is a radiofrequency balloon (RFB) for PVI (Heliostar, Biosense Webster™) designed to deliver single-shot circumferential pulmonary vein isolation. The system is based on a 28-mm compliant and irrigated balloon catheter that can be visualized in a 3D mapping platform (Carto, Biosense Webster™). Complementary to this, a circular mapping catheter (Lassostar, Biosense Webster™) can be introduced via the inner lumen to guide the balloon, to record pulmonary vein potentials, and to perform voltage mapping. The operator can evaluate the quality of the energy applications though real-time monitoring of impedance and temperature values [5] from the 10 surface electrodes and selectively titrate the energy delivered (Fig. 1).

Recent studies [6–8] have reported data on acute efficacy and safety of this system in obtaining PVI. Apart from the first-in-man RADIANCE [6] and SHINE [7] studies, which encompassed only patients with paroxysmal atrial fibrillation (PAF), long-term rhythm outcome reports after PVI using this system are lacking.

Two high-volume German ablation centers have collaboratively contributed to a prospective registry (AURORA), where consecutive patients with PAF or persistent AF (Pers-AF) undergoing RFB-based PVI were enrolled. All patients included in the AURORA registry underwent a structured follow-up at 90, 180, and 360 days post-ablation. In this study, we report the long-term safety and efficacy outcomes of RFB-PVI and analyzed potential predictors of late AF recurrence.

## Methods

### Inclusion and exclusion criteria

Two high-volume German centers (University Heart and Vascular Center Hamburg Eppendorf – Hamburg, Germany and Cardioangiologisches Centrum Bethanien – Frankfurt am Main, Germany) shared their experience using the RFB in the AURORA collaboration. Consecutive patients were enrolled in a prospective registry [9]; we now report on a subgroup of them that agreed to undergo a structured and monitored long-term follow-up, including clinical re-evaluation and 48-h Holter ECGs at 90, 180, and 360 days following ablation.

**Fig. 2:**
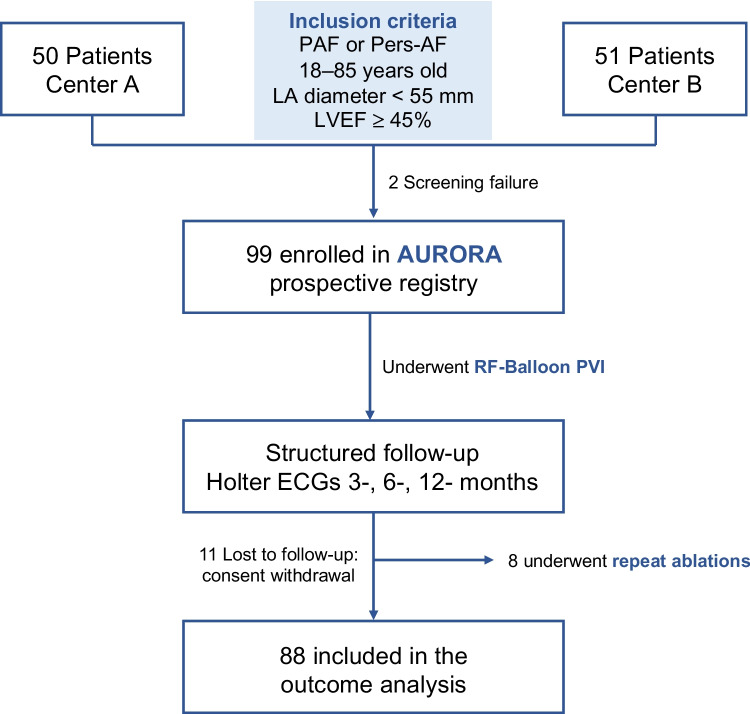
Flow chart of the study

Inclusion criteria were non-valvular AF, age between 18 and 85 years, a left atrial size < 55 mm, and a left ventricular ejection fraction (LVEF) ≥ 45%. Exclusion criteria consisted in any contraindications to oral anticoagulation (OAC), presence of intracardiac thrombi, pregnancy, participation in other clinical studies, mitral valve regurgitation or stenosis higher than moderate, history of mitral valve surgery, previous left atrial appendage ligation or closure, and obstructive sleep apnea. All patients provided written informed consent, and the study was approved by the institutional review board (2020–2191-evBO) (Fig. 2).

### Ablation procedure

Catheter ablation was performed under deep sedation using midazolam, fentanyl, and propofol. Two femoral venous accesses were obtained: one to advance a 7F steerable decapolar catheter (Parahis, Biosense Webster) into the coronary sinus and the second for single transeptal puncture using a modified Brockenbrough technique and a SL1 sheath (8.5F, St. Jude Medical). After transseptal puncture, intravenous heparin was administered to maintain an activated clotting time (ACT) > 300 s.

PV ostia were identified according to selective PV angiograms. Pre- and post-ablation 3D mapping of the LA was obtained via the LASSO NAV catheter (Biosense Webster). After LA mapping, the regular transseptal sheath was changed over-the-wire for the Guidestar sheath (14F inner diameter). RFB positioning at the PV ostium was performed using both fluoroscopic and 3D mapping guidance. Real-time PV electrograms were obtained by the Lassostar catheter and the RFB electrodes (Fig. 1).

Impedance and temperature values for each RFB electrode were displayed in real time and indicated balloon-tissue contact. In order to provide optimal electrode-tissue contact and RFB positioning, the following parameters were targeted: inflation index > 0.8, impedance of 100 ± 20 Ohms across all electrodes, and temperature variability across all electrodes < 3 °C, with a maximum temperature of 31 °C. RFB applications were delivered at a temperature-controlled unipolar energy mode of 15 W, and a target electrode temperature during RF delivery of 55 °C. As recommended by the manufacturer, each application lasted 60 seconds (s) for the electrodes facing the anterior, superior, and inferior aspects of the targeted PV and 20–30 s for electrodes facing the posterior wall. Energy delivery was immediately stopped along the posterior wall electrodes at esophageal temperature rise > 39 °C as measured by an esophageal temperature probe (Circa S-Cath™, Bisping Medizintechnik GmbH or SensiTherm, St. Jude Medical, St. Paul, MN, USA). If the esophageal temperature further increased, the electrodes adjacent to the posterior electrodes were also switched off.

Before ablation of the right-sided PVs, pacing of the anterior electrodes at maximal output (2 ms, 10 mA) was performed to unmask potential phrenic nerve capture at the desired balloon position. If phrenic nerve capture was observed, the catheter was repositioned and inflation flow of the balloon was adapted, and pacing from the anterior electrodes was repeated to confirm the absence of phrenic nerve capture at the new location. During RFB applications along the right-sided PVs, phrenic nerve pacing was performed from a decapolar catheter positioned in the superior vena cava at a cycle length of 700 ms and at maximal output. Ablation was stopped instantaneously if phrenic nerve capture was weakened or lost.

### Definition of complications

Major complications were defined as air embolism, pericardial tamponade, bleeding resulting in hemorrhagic shock and requiring catecholamines, high-degree (≥ 50%) PV stenosis, phrenic nerve palsy, transient ischemic attack (TIA), stroke, infection, myocardial infarction, atrio-esophageal fistula, and death. Hematoma at access sites, pericardial effusion (> 5 mm) and PV stenosis (< 50%) were considered minor complications.

### Clinical follow-up

The first clinical FU post-ablation was performed on the day of hospital discharge. Subsequently, the patients completed routine outpatient clinic visits at 90, 180, and 360 days post-ablation. Each visit included a 48-h Holter ECG. Additional telephone interviews and/or outpatient clinic visits were initiated if patients reported symptoms suggestive of arrhythmia recurrence. Any documented episode of atrial tachyarrhythmia lasting > 30 s was considered as recurrence.

### Statistical analysis

All categorical variables, such as patient and procedural characteristics, are reported as absolute and relative frequencies. The continuous variables were tested for normal distribution using the Shapiro–Wilk test. They were reported as mean ± standard deviation in case of normal distribution and as median and interquartile range (IQR) (first quartile, third quartile) otherwise. The continuous variables were compared using the non-paired Student’s *t*-test when normally distributed and the non-parametric tests (Mann–Whitney *U* test or Kruskal–Wallis) otherwise. Multivariate analysis to identify predictors of AF recurrence was performed using Cox regression analysis and was reported as odds ratio (OR) and 95% confidence intervals (CIs). All *p* values are two sided. A *p* value of < 0.05 was considered significant. Data were summarized in an Excel sheet and statistical analyses were performed using GraphPad Prism versions 8 and 10.

## Results

### Baseline patient characteristics

A total of 99 patients (50 from Center A, 49 from Center B) undergoing PVI for PAF or Pers-AF were enrolled in the study (Fig. 2). In total, 43/99 (43%) patients were women, median age was 67 years (IQR 59–74), and median LA diameter was 41 mm (IQR 37–47). Median LVEF was 60 (IQR 62–55) and median CHA_2_DS_2_-VASc score was 3 (mean 3.3 ± 1.5). A total of 54/99 (54.5%) patients presented with PAF, 43/99 (43.4%) patients with Pers-AF. The mean duration of AF since first diagnosis was 2.6 ± 3 years (Table 1).

**Table 1:** Baseline patient characteristics

Baseline characteristics	*N* = 99
Female sex, *n* (%)	43 (43.4)
Age, median (Q1–Q3) (years)	67 (59–74)
Body mass index, median (Q1–Q3) (kg/m^2^)	26.8 (24.8–31.1)
Persistent AF, *n* (%)	43 (43.4)
Arterial hypertension, *n* (%)	63 (63.6)
Diabetes mellitus, *n* (%)	15 (15.2)
Stroke, *n* (%)	4 (4)
Coronary artery disease, *n* (%)	16 (16.2)
LA diameter, median (Q1–Q3) (mm)	41 (37–47)
LVEF, median (Q1–Q3) (%)	60 (55–62)
Failure of > 1 AAD, *n* (%)	44 (44.4)
Sodium channel blockers, *n* (%)	8 (8)
Potassium channel blockers, *n* (%)	11 (11.1)
Beta-blockers, *n* (%)	62 (62.6)

*AAD* antiarrhythmic drugs, *AF* atrial fibrillation, *LA* left atrial, *LVEF* left ventricular ejection fraction, *Q1* first quartile, *Q3* third quartile

### Procedural data and ablation results

In all 99 patients, successful PVI was performed as described above. A total of 383 PVs were identified (5 LCPV, 1 RCPV, 1 RMPV) and all (100%) PVs could be acutely isolated using solely the RFB; no touch-up focal ablations were required. Of note, 211/383 (55.1%) PVs were isolated with a single-shot RF application. The overall median skin-to-skin procedure time was 67 min (IQR 57–85) and the median LA dwell time was 49 (IQR 39–62) min. Median RF ablation time was 23 (IQR 18–32) min. The median fluoroscopy time was 12 min (IQR 9.3–15.2) with the median dose area product 761 (IQR 509–1534) uGycm^2^. Esophageal temperature > 39 °C was recorded in 59/383 (15%) PVs and in 41/99 (41%) patients, most commonly at the LIPV followed by RIPV ((20% and 19%, respectively), LSPV (15%), and RSPV (5%)). The mean maximum temperature per vein was 37.5 °C ± 2.6 °C for the LSPV, 38.1 °C ± 2.6 °C for the LIPV, 36.9 °C ± 1.6 °C for the RSPV, and 38 °C ± 2.7 °C for the RIPV (Table 2).

**Table 2:** Procedural data

Procedural characteristics	*N* = 99
Procedure time, median (Q1–Q3) (min)	67 (57–85)
Fluoroscopy time, median (Q1–Q3) (min)	12 (9.3–15.2)
Fluoroscopy dose, median (Q1–Q3) (uGycm^2^)	761 (509–1534)
Total PVs, *n*	383
PVI acute success (%)	383/383 (100%)
Single shot isolations, *n* (%)	211/383 (55.1%)
LA time, median (Q1–Q3) (min)	49 (39–62)
Ablation time, median (Q1–Q3) (min)	23 (18–32)
Esophageal temperature > 39 °C (%)	59/383 (15%)

*LA* left atrial, *PVI* pulmonary vein isolation, *PVs* pulmonary veins, *Q1* first quartile, *Q3* third quartile

**Fig. 3:**
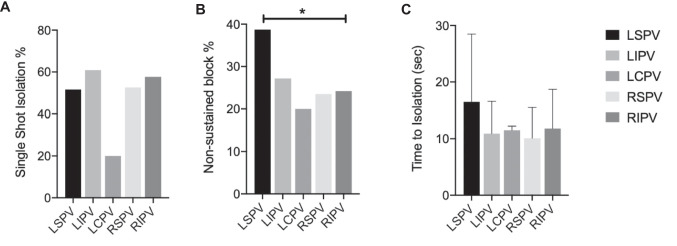
**A**–**C** Ablation data per pulmonary vein. Abbreviations: LCPV, left common pulmonary vein; LIPV, left inferior pulmonary vein; LSPV, left superior pulmonary vein; RIPV, right inferior pulmonary vein; RSPV, right superior pulmonary vein; TTI, time to isolation

Excluding the LCPVs, the LSPVs showed the lowest rate of single-shot isolation (51.6% when compared to the LIPVs (60.9%), RSPVs (52.6%), and RIPVs (57.7%) (Table 3, Fig. 3A)).

**Table 3:** Ablation data per PV

LSPV	Single-shot isolation	48/93 (51.6)
	TTI, median (s)	12 (IQR 8.7–17)
	Non-sustained block	36/93 (38.7)
LIPV	Single-shot isolation	56/92 (60.9)
	TTI, median (s)	9 (IQR 7–11.7)
	Non-sustained block	25/92 (27.2)
LCPV	Single-shot isolation	1/5 (20)
	TTI, median (s)	11.5 (IQR 11.2–11.7)
	Non-sustained block	1/5 (20)
RSPV	Single-shot isolation	50/95 (52.6)
	TTI, median (s)	8 (IQR 7–11)
	Non-sustained block	20/85 (23.5)
RIPV	Single-shot isolation	56/97 (57.7)
	TTI, median (s)	10 (IQR 8–13.7)
	Non-sustained block	23/95 (24.2)

*IQR* interquartile range, *LCPV* left common pulmonary vein, *LIPV* left inferior pulmonary vein, *LSPV* left superior pulmonary vein, *RIPV* right inferior pulmonary vein, *RSPV* right superior pulmonary vein, *TTI* time to isolation

In case of single-shot PVI, median time to isolation (TTI) was 10 s (IQR 8–14), with the LSPV requiring longer TTI (12 s, IQR 8.7–16.1) compared to the other PVs (LIPV 9 s, IQR 7–11.7; RSPV 8 s, IQR 7–11; and RIPV 10 s, IQR 8–13.5), without reaching statistical significance due to high variability in the group (Table 3, Fig. 3C). Parallelly, the LSPV showed a higher rate of non-sustained block when compared to all other PV, in particular to RIPV (*p* = 0.01) (Table 3, Fig. 3B).

### Safety

In the present patient cohort, safety events were recorded in 3/99 (3%) patients (Table 4). We recorded one acute procedural safety event and two long-term events. One patient presented with pharyngeal bleeding 1 day after discharge in the absence of significant hemoglobin reduction requiring transfusion. Importantly, this patient underwent preprocedural transesophageal echocardiography. One patient presented with NSTEMI 7 months after the PVI procedure. One patient died of non-cardiac cause 5 months after the procedure. No other serious adverse such as air embolism, TIA/stroke, or tamponade occurred. No patient presented intraprocedural phrenic palsy. In our experience, phrenic nerve capture was more likely in patients with larger right superior pulmonary veins, although the exact number of cases was not systematically recorded. Ablation was always initiated only at a site where no capture was observed under baseline conditions, and in our cohort, no patients experienced a reduction or loss of capture during radiofrequency application. No patient reported symptoms attributable to esophageal damage during the follow-up. Minor complications were not observed.

**Table 4:** Safety events

Safety events	
Air embolism, *n* (%)	0
Bleeding, *n* (%)	1/99 (1%)
PV stenosis, *n* (%)	0
Pericardial effusion, *n* (%)	0
Tamponade, *n* (%)	0
Phrenic palsy, *n* (%)	0
Stroke, *n* (%)	0
TIA, *n* (%)	0
Infection, *n* (%)	0
MI, *n* (%)	1/99 (1%)
Non-cardiac death, *n* (%)	1/99 (1%)
Access site complications, *n* (%)	0

*MI* myocardial infarction, *PV* pulmonary vein, *TIA* transient ischemic attack

### Follow-up

**Fig. 4:**
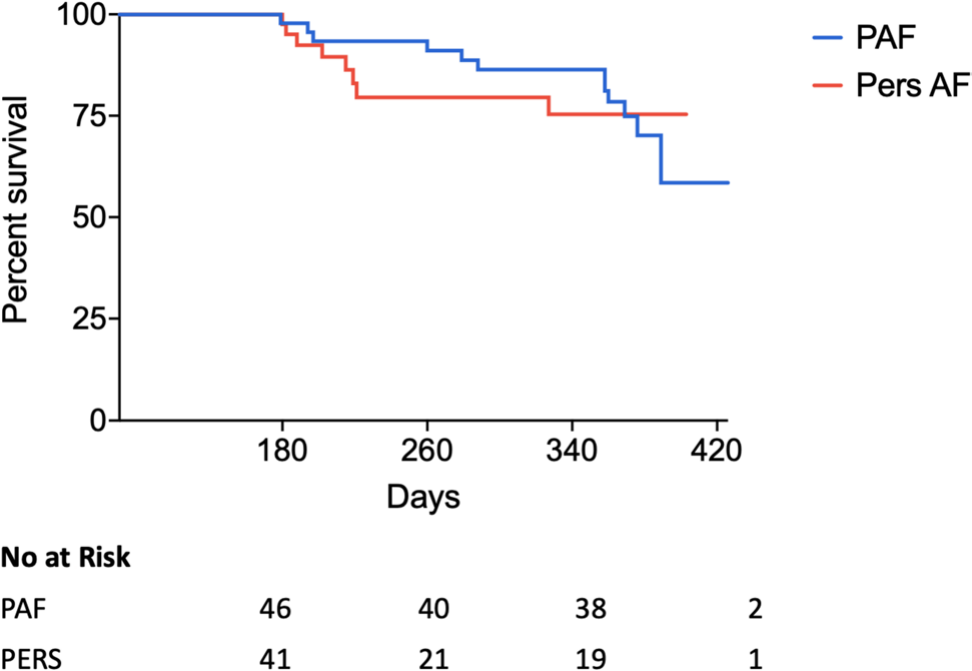
Long-term follow-up. Kaplan–Meier survival analysis after the blanking time. Abbreviations: PAF, paroxysmal atrial fibrillation; Pers-AF, persistent atrial fibrillation

Among 99 patients included in this study, 11 (11.1%) were lost to FU. Eighty-eight patients (49 (55.7%) male, age 65 years (IQR 58.5–10.5), LA diameter 42 mm (IQR 34–46)) completed the structured follow-up and were included in the analysis. The overall median FU duration was 361 (IQR 261–375) days. Kaplan–Meier estimated freedom of AF/AT out of the blanking time was 78.4% for PAF and 75.4% for Pers-AF (*p* = 0.828) at 1 year (Fig. 4). During a 90-day blanking period, early recurrence of atrial tachyarrhythmias (ERAT) had occurred in 27/88 (30.7%) patients, with 13/27 (48.2%) of these patients presenting with Pers-AF. Among the patients presenting with ERAT, 14/27 (51.8%) had Pers-AF at baseline.

### Repeat ablations

**Fig. 5:**
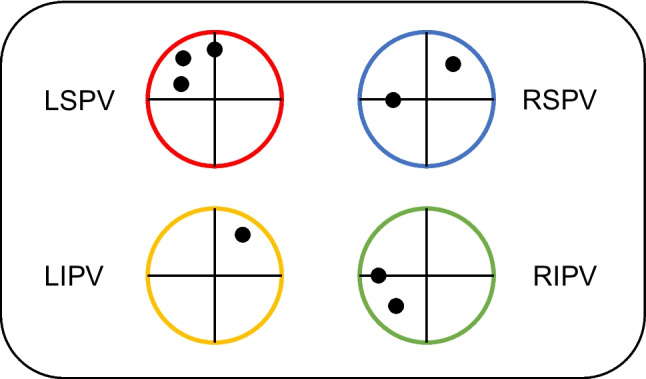
Localization of electrical reconduction gaps in repeat procedures. Graphical presentation of electrical reconduction gaps observed as assessed during repeat ablation procedures. Abbreviations: MI, myocardial infarction; PV, pulmonary vein; TIA, transient ischemic attacks

A repeat ablation procedure was performed in 8/99 (8%) patients during the course of the study. Six patients presented with AF, 2 with atypical atrial flutter. Repeat ablation was performed after a median of 216 (IQR 193.5–308.5) days following the index RFB procedure. The index arrhythmia was Pers-AF in 5/8 (62.5%) patients. Overall, durable isolation was documented in 25/32 (78%) PVs. No PV stenosis or narrowing was recorded. Three patients presented all PV isolated and a substrate modification strategy was adopted. The localization of the documented PV reconnection gaps is shown in Fig. 5.

### Predictors of recurrent AF

To explore potential predictors of AF recurrence after the blanking period, a multiple regression analysis was conducted, considering various clinical and procedural factors such as sex, age, BMI, left atrial diameter, LVEF, ablation time, procedure time, single-shot PVI, and ERAT. In this analysis, ERAT emerged as the only independent predictor for AF recurrence at 1 year, with a hazard ratio of 3.198 and a 95% confidence interval of 1.036 to 10.32 (*p* value = 0.0433) (Table 5).

**Table 5:** Predictors of arrhythmia recurrence

	HR	95% CI	*p* value
Female sex	0.2851	0.06734 to 1.045	0.0693
Age	1.022	0.9598 to 1.092	0.5092
BMI	1.057	0.9430 to 1.179	0.3208
LA	0.9278	0.8397 to 1.016	0.1181
LVEF	1.036	0.9814 to 1.102	0.2185
Ablation time	0.9808	0.8986 to 1.074	0.6622
LA time	1.020	0.9249 to 1.101	0.6529
Procedure time	0.9656	0.8898 to 1.034	0.3547
All single shot	0.5149	0.1403 to 1.784	0.2989
Recurrence in blanking	3.198	1.036 to 10.32	0.0433

*BMI* body mass index, *LA* left atrial diameter, *LVEF* left ventricular ejection fraction

## Discussion

In a multicenter cohort of patients with PAF and Pers-AF, PVI with the novel RFB catheter was safe, efficient, and resulted in a high rate of freedom from recurrent atrial arrhythmias. ERAT was the only predictor of late recurrence (Central Illustration).

### One-year outcome

This study represents the first multicenter real-word prospective analysis of acute and long-term outcome of patients undergoing RFB-based PVI. In this cohort of patients, RFB-PVI was performed with a 100% acute success rate; the Kaplan–Meier estimates for freedom from atrial tachyarrhythmias were 78.4% for PAF and 75.4% for Pers-AF. This success rate is slightly higher when compared to the SHINE study [7] that evaluated the outcome of RF-PVI in a group of patients with PAF only. Our results are also comparable to other established and emerging energies: in the recent ADVENT trial, modern RF point-by-point ablation, cryoballoon ablation, and the newly introduced pulsed field ablation led to a 1-year follow-up in paroxysmal AF between 71 and 73% [8].

The favorable long-term clinical outcomes were achieved by considerably short procedure times with a median procedure time of 67 min, and a median LA time of only 47.5 min. These data are comparable to data published about the well-established 4th generation cryoballoon [10], but also with the average procedural time recorded with a single-shot PFA device in a large European registry [11].

The rate of single-shot PVI is in line with what has been observed in acute efficacy studies [9–12]. However, contrary to our expectations, PVI obtained by single-shot RF applications did not impact long-term FU outcomes.

The LSPV, as previously described [5, 9], presented the highest rate of non-sustained block and lowest rate of single-shot isolation. Accordingly, the LSPV was also the PV with the highest rate of electrical reconduction during repeat ablation procedures in patients with AF recurrences. This might be reflected by the frequently challenging anatomy of this specific PV making it more difficult to obtain perfect circumferential balloon-to-PV contact and thus optimal balloon-tissue energy transfer.

### Single-shot catheter integrated in a 3D platform

Single-shot devices are designed to simplify PVI procedures, but they often rely primarily on fluoroscopy guidance. This system, however, was launched with integrated 3D mapping, which enhances catheter visualization, positioning at the PV ostium, and navigation in the LA, especially compared to other balloon techniques. Impedance and temperature values can be used as a surrogate of circumferential contact of the balloon with the PV ostium, without the need for occlusion angiograms [5]. The integration into 3D mapping provides additional information on the electrical substrate of the left atrium, while reducing radiation exposure. Moreover, the system enables real-time monitoring of ablation parameters at the side of the 3D mapping (Fig. 1). By continuously confirming tissue contact and recording electrograms, the RF balloon provides feedback to the operator, enabling precise energy titration and selective ablation in cases of electrical gaps. Consequently, the catheter’s position can be adjusted based on the information obtained. Although the associated costs can be in specific settings high, the potential benefits may justify the additional expense in selected clinical scenarios.

### Safety profile

In the current series, RFB-PVI was safe, with a complication rate in line with other technologies, like cryoballoon or point-by-point RF-PVI [13]. The cumulative rate of safety events during FU was 3%, consisting of only 1 non-serious bleeding, 1 NSTEMI, and 1 death of non-cardiac cause. The favorable safety profile observed can be attributed to the benefits of an over-the-wire (Lasso) balloon technology allowing for safe manipulation and maneuvering inside the LA. In addition, with the energy levels selected (15 W × 60 s), we have never recorded steam pops. There were no further major safety events during follow-up, including no phrenic nerve palsy, PV stenosis, or TIA/stroke.

In the present structured follow-up, no patients experienced dysphagia or other symptoms suggestive of severe esophageal involvement. However, in this study, routine post-ablation endoscopy was not performed. RF energy delivery at the posterior portion of the PV antrum may lead to esophageal lesions, and in the worst-case scenario to esophageal fistulas. At the time of writing this manuscript, an urgent field safety notice has been issued by the manufacturer concerning atrio-esophageal fistulas. In our previous work on acute efficacy and safety of this technology, an esophageal endoscopy was performed in unselected 85 patients after a median of 1 day after the procedure. Seven thermic endoscopy-detected esophageal lesions (EDEL) (8%) were recorded, all consisting in ulcers of 0.2–1 cm in diameter. The biggest ulcer was described in a patient with a marked temperature rise (47 °C) during ablation at the LIPV, when the esophageal probe acoustic alarm did not work properly. A control endoscopy after 4 weeks revealed an almost complete healing of this lesion. Six out of seven patients (86%) with EDEL had a temperature rise in the esophagus, vs. 31/78 (40%) patients without EDEL. Patients with EDEL were in trend treated with more applications at the left-sided PVs (7 ± 3 vs. 4 ± 3, *p* = 0.07), with significantly more applications at the LSPV (5 ± 3 vs. 2 ± 2, *p* = 0.04) compared to patients without EDEL. Procedural time in patients with EDEL was significantly longer [9].

The risk of esophageal lesions may be avoided by a new non-thermal energy source as the PFA. Future evolution of the RFB technology maybe enabling a toggling between RFC and PFA could further increase the safety and efficacy profile of this platform.

### Early recurrences predict late outcome

ERAT are demonstrated in almost every third patient after RFB-PVI at the first FU visit. The early pro-arrhythmogenic effect of RF ablation has been largely discussed in literature and relates to ischemia, coagulation necrosis, edema, and local inflammation of atrial tissue [14, 15]. Non-optimal energy transfer at specific PVs and specific aspects of the PVs might result in sub-optimal energy transfer and thus potentially in early electrical reconduction into acutely isolated PVs. ERAT has been described as a predictor of late recurrences after point-by-point RF ablation and for cryoablation [16–18]. Our data underlie the fact that, also for the novel RFB, ERAT are prognostically important for the long-term outcome of PVI procedures. These findings question the practice of ignoring AF recurrences in the first 90 days after AF ablation (“blanking period”).

Other factors that are used to identify patients at high risk of recurrent AF, e.g., enlarged left atria, persistent AF, or a long duration of AF prior to AF ablation [1, 19, 20], were not associated with recurrent AF in this study. While our analysis, limited to 99 patients, cannot rule out an effect of these parameters on recurrent AF, such effects were weak in our data set.

## Conclusion

In a multicenter cohort of patients with PAF and Pers-AF, PVI with the novel RFB catheter was safe and efficient, and resulted in a high rate of freedom from recurrent atrial arrhythmias. Early recurrences of atrial arrhythmias were the only predictor of recurrent AF.

## References

[1] Hindricks G, Potpara T, Dagres N, et al. Corrigendum to: 2020 ESC Guidelines for the diagnosis and management of atrial fibrillation developed in collaboration with the European Association for Cardio-Thoracic Surgery (EACTS): The Task Force for the diagnosis and management of atrial fibrillation of the European Society of Cardiology (ESC) Developed with the special contribution of the European Heart Rhythm Association (EHRA) of the ESC. Eur Heart J. 2021;42(40):4194. 10.1093/eurheartj/ehab648. 34520521 10.1093/eurheartj/ehab648

[2] Kirchhof P, Camm AJ, Goette A, et al. Early rhythm-control therapy in patients with atrial fibrillation. N Engl J Med. 2020;383(14):1305–16. 10.1056/NEJMoa2019422. 32865375 10.1056/NEJMoa2019422

[3] Packer DL, Mark DB, Robb RA, et al. Effect of catheter ablation vs antiarrhythmic drug therapy on mortality, stroke, bleeding, and cardiac arrest among patients with atrial fibrillation: the CABANA Randomized Clinical Trial. JAMA. 2019;321(13):1261–74. 10.1001/jama.2019.0693. 30874766 10.1001/jama.2019.0693 PMC6450284

[4] Metzner A, Kuck KH, Chun JKR. What we have learned: is pulmonary vein isolation still the cornerstone of atrial fibrillation ablation?. Europace. 2022;24(Suppl 2):ii8-ii13. 10.1093/europace/euab268 10.1093/europace/euab268 35661870

[5] My I, Bordignon S, Butt M, et al. Novel radiofrequency balloon catheter - impact of ablation parameters on single-shot isolation. Circ J. 2023;87(7):950–6. 10.1253/circj.CJ-23-0220. 37286488 10.1253/circj.CJ-23-0220

[6] Reddy VY, Schilling R, Grimaldi M, et al. pulmonary vein isolation with a novel multielectrode radiofrequency balloon catheter that allows directionally tailored energy delivery: short-term outcomes from a multicenter first-in-human study (RADIANCE). Circ Arrhythm Electrophysiol. 2019;12(12): e007541. 10.1161/CIRCEP.119.007541. 31826648 10.1161/CIRCEP.119.007541

[7] Schilling R, Dhillon GS, Tondo C, et al. Safety, effectiveness, and quality of life following pulmonary vein isolation with a multi-electrode radiofrequency balloon catheter in paroxysmal atrial fibrillation: 1-year outcomes from SHINE. Europace. 2021;23(6):851–60. 10.1093/europace/euaa382. 33450010 10.1093/europace/euaa382 PMC8186540

[8] Reddy VY, Gerstenfeld EP, Natale A, et al. Pulsed field or conventional thermal ablation for paroxysmal atrial fibrillation. N Engl J Med. 2023;389(18):1660–71. 10.1056/NEJMoa2307291. 37634148 10.1056/NEJMoa2307291

[9] Bordignon S, My I, Tohoku S, et al. Efficacy and safety in patients treated with a novel radiofrequency balloon: a two centres experience from the AURORA collaboration. Europace. 2023;25(5):euad106. 10.1093/europace/euad106. 37116126 10.1093/europace/euad106 PMC10228597

[10] Straube F, Dorwarth U, Pongratz J, et al. The fourth cryoballoon generation with a shorter tip to facilitate real-time pulmonary vein potential recording: feasibility and safety results. J Cardiovasc Electrophysiol. 2019;30(6):918–25. 10.1111/jce.13927. 30907462 10.1111/jce.13927

[11] Schmidt B, Bordignon S, Neven K, et al. EUropean real-world outcomes with Pulsed field ablatiOn in patients with symptomatic atRIAl fibrillation: lessons from the multi-centre EU-PORIA registry. Europace. 2023;25(7):euad185. 10.1093/europace/euad185. 37379528 10.1093/europace/euad185 PMC10320231

[12] Del Monte A, Almorad A, Pannone L, et al. Pulmonary vein isolation with the radiofrequency balloon catheter: a single centre prospective study. Europace. 2023;25(3):896–904. 10.1093/europace/euad017. 36738245 10.1093/europace/euad017 PMC10062286

[13] Wu C, Li X, Lv Z, et al. Second-generation cryoballoon versus contact force radiofrequency ablation for atrial fibrillation: an updated meta-analysis of evidence from randomized controlled trials. Sci Rep. 2021;11(1):17907. 10.1038/s41598-021-96820-8. (Published 2021 Sep 9). 34504121 10.1038/s41598-021-96820-8 PMC8429450

[14] Gottlieb LA, Dekker LRC, Coronel R. The blinding period following ablation therapy for atrial fibrillation: proarrhythmic and antiarrhythmic pathophysiological mechanisms. JACC Clin Electrophysiol. 2021;7(3):416–30. 10.1016/j.jacep.2021.01.011. 33736761 10.1016/j.jacep.2021.01.011

[15] Mugnai G, de Asmundis C, Hünük B, et al. Second-generation cryoballoon ablation for paroxysmal atrial fibrillation: predictive role of atrial arrhythmias occurring in the blanking period on the incidence of late recurrences. Heart Rhythm. 2016;13(4):845–51. 10.1016/j.hrthm.2015.12.034. 26724490 10.1016/j.hrthm.2015.12.034

[16] Steinberg C, Champagne J, Deyell MW, et al. Prevalence and outcome of early recurrence of atrial tachyarrhythmias in the Cryoballoon vs Irrigated Radiofrequency Catheter Ablation (CIRCA-DOSE) study. Heart Rhythm. 2021;18(9):1463–70. 10.1016/j.hrthm.2021.06.1172. 34126269 10.1016/j.hrthm.2021.06.1172

[17] Calkins H, Gache L, Frame D, et al. Predictive value of atrial fibrillation during the postradiofrequency ablation blanking period. Heart Rhythm. 2021;18(3):366–73. 10.1016/j.hrthm.2020.11.020. 33242668 10.1016/j.hrthm.2020.11.020

[18] Vrachatis DA, Papathanasiou KA, Kossyvakis C, et al. Early arrhythmia recurrence after cryoballoon ablation in atrial fibrillation: a systematic review and meta-analysis. J Cardiovasc Electrophysiol. 2022;33(3):527–39. 10.1111/jce.15337. 34951496 10.1111/jce.15337

[19] Dretzke J, Chuchu N, Agarwal R, et al. Predicting recurrent atrial fibrillation after catheter ablation: a systematic review of prognostic models. Europace. 2020;22(5):748–60. 10.1093/europace/euaa041. 32227238 10.1093/europace/euaa041 PMC7203634

[20] Joglar JA, Chung MK, Armbruster AL, et al. 2023 ACC/AHA/ACCP/HRS Guideline for the diagnosis and management of atrial fibrillation: a report of the American College of Cardiology/American Heart Association Joint Committee on Clinical Practice Guidelines [published correction appears in Circulation. 2024 Jan 2;149(1):e167]. Circulation. 2024;149(1):e1-e156. 10.1161/CIR.0000000000001193. 10.1161/CIR.0000000000001193 PMC11095842 38033089

